# Pharmacological use of gamma-aminobutyric acid derivatives in osteoarthritis pain management: a systematic review

**DOI:** 10.1186/s41927-022-00257-z

**Published:** 2022-05-11

**Authors:** Ze Du, Hanxiao Chen, Yongrui Cai, Zongke Zhou

**Affiliations:** 1 Department of Orthopedics, Research Institute of Orthopedics, West China Hospital/West China School of Medicine, Sichuan University, Chengdu, 610041 China; 2 Department of Obstetrics and Gynaecology, West China Second University Hospital, Sichuan University, Chengdu, 610041 China; 3 Key Laboratory of Birth Defects and Related Diseases of Women and Children of the Ministry of Education, West China Second University Hospital, Sichuan University, Chengdu, 610041 China

**Keywords:** Osteoarthritis, GABA derivatives, Pain management, Systematic review

## Abstract

**Background:**

Pain is the major complication of osteoarthritis (OA) patients and is a decisive symptom for medical intervention. Gamma-aminobutyric acid (GABA) derivatives are optional painkillers but not widely used in pain management of OA patients. We synthesized the efficacy and safety of GABA derivatives for OA pain management.

**Methods:**

We searched Medline, Cochrane CENTRAL, Embase, and ClinicalTrals.gov from inception to 13 October 2021 and included randomized controlled trials (RCTs) comparing the efficacy and safety of GABA derivatives with placebo or standard control in OA pain management. Two independent reviewers extracted data and assessed these studies for risk of bias using Cochrane Collaboration’s tool for RCT.

**Results:**

In total, three eligible RCTs (n = 3) meeting the eligibility criteria were included. Among these RCTs, one focused on hand OA pain management, while two RCTs focused on knee OA. In hand OA, pregabalin reduced numerical rating scale (NRS) score and the Australian/Canadian Osteoarthritis Hand Index (AUSCAN) pain score significantly compared with placebo, and caused 55 AEs. In knee OA, pregabalin reduced visual analogue scale (VAS) score and the Western Ontario and McMaster Universities Arthritis Index (WOMAC) pain score significantly with no recorded adverse event (AE). Meanwhile, in knee OA, gabapentin reduced both VAS score and WOMAC pain score compared with acetaminophen and caused 9 AEs.

**Conclusions:**

GABA derivatives seem to be effective and safe in OA pain management. However, future researches with large sample size are needed to further prove the efficacy of GABA derivatives in OA pain control.

*Trial registration*: CRD42021240225.

**Supplementary Information:**

The online version contains supplementary material available at 10.1186/s41927-022-00257-z.

## Background

Osteoarthritis (OA) is considered as an aging and chronic disease. Concurrently, more than 250,000,000 people in the world are burdened with OA, which makes OA the leading cause of disability and cost of social sources [1].

Chronic pain is the most common symptom in OA patients, and is the main reason for patients to seek medical attention. Also, pain is one of the decisive symptoms of whether medical intervention is needed [2]. The typical pain of knee OA is chronic, intermittent, and related to abnormal mechanical loading. With the progression of OA, pain can be more frequent, more severe, more persistent, and more unacceptable [3]. The generation of pain in OA involves various complex mechanisms. In general, OA is considered as an articular disease with local tissue injury or inflammation. This activates the peripheral nociceptors around the OA joint, which causes nociceptive pain [4]. Moreover, the chronic pain in OA can be explained by neuropathic and central mechanisms [1]. Neuropathic pain is caused by aberrant innervation in OA joint and damage in sensory neurons [5]. Central pain is associated with hyperexcitability in central nervous system in OA patients, which can be more refractory [6].

To manage pain in OA patients, multiple pharmacological therapies are utilized, targeting joint inflammation, nociceptive pathways, and neuropathic and central pathways. For nociceptive pain, anti-inflammatory drugs, including nonsteroidal anti-inflammatory drugs (NSAIDs) and glucocorticoids are significantly efficient, owing to its scavenging action of inflammatory cytokines [7, 8]. Meanwhile, antibody of neurotrophins, for example, anti-nerve growth factor (NGF) antibody, is also able to relieve nociceptive pain [9]. However, there are challenges of neuropathic and central pain management, owing to complicated nerve pathological changes. Although duloxetine, a serotonin and norepinephrine reuptake inhibitor, can work as an analgesic to reduce central pain in OA patients, the most effective drug to release neuropathic and central pain is still elusive [10].

Gamma-aminobutyric acid (GABA) derivatives are known as adjunctive medication for antiepileptic drugs. GABA derivative mainly includes gabapentin, pregabalin, baclofen, and vigabatrin. In general, gabapentin is a sodium (Na) channel blocker. With an appropriate dose, gabapentin can reduce abnormal impulses in injured neurons, and release neuropathic pain [11]. In central nervous system, gabapentin can bind to the α2*δ* subunit of voltage-gated calcium (Cav) channels to block such calcium channels, and depress central hypersensitivity to pain [12]. Similar to gabapentin, pregabalin also binds to α2*δ* subunit of Cav channels and releases central pain [13]. In that case, these analgesic agents are likely to release OA pain.

Here, we performed this systematic review to investigate the efficacy and safety of GABA derivatives in OA pain relief and to provide a new insight into personalized OA pain management.

## Methods

### Search strategy

This systematic review was registered on PROSPERO (CRD42021240225). We systematically searched Medline, Cochrane CENTRAL, Embase, and ClinicalTrals.gov from the earliest record to 13 October 2021. Only the studies written in English were included. The following terms were utilized in literature search: OA, GABA, gabapentinoids, anticonvulsants, pregabalin, gabapentin, baclofen, vigabatrin. A full list of search strategy is provided in Additional file 1: Appendix S1. Two reviewers, Yongrui Cai and Ze Du, independently screened the titles, abstracts and full-texts, and confirmed eligible studies. When these two reviewers had a disagreement, then another reviewer, Hanxiao Chen, made the final decision.

### Eligibility criteria

Inclusive criteria for eligible literatures were: (a) randomized controlled trails (RCTs) that compared GABA derivatives versus placebo and any other medicine or combined use of GABA derivatives and any other medicine versus such specific medicine and placebo in participants with OA; (b) unpublished data on clinical trial registry platforms; (c) studies considering joint pain and disability as an outcome; (d) literatures in English language.

Exclusive criteria were: (a) RCT that focused on GABA derivatives treatment for perioperative analgesia of OA; (b) studies involved rheumatoid arthritis (RA) participants.

### Data extraction

We extracted data from eligible studies including study characteristics, basic information of participants, details of GABA derivative use, reported pain and function outcomes, safety data, and author’s conclusion. For study characteristics, we abstracted following issues: study name, publish year, published journal, the sample size, study design. For basic information of participants, we extracted following data: nation, age range of participants, the female ratio, specific joint with OA, mean duration of OA, and severity of OA. For details of GABA derivatives use, we extracted following data: the specific name, usage and dose, duration of drug use of GABA derivatives, combination medicine, and controlled medicine. For outcomes, we abstracted following issues: Western Ontario and McMaster Universities Arthritis Index (WOMAC) pian score, Visual analogue scale (VAS) score, Australian and Canadian Hand Osteoarthritis Index (AUSCAN) pain score, and Numerical Rating Scale (NRS) score. The safety data included the frequency of recorded treatment-related adverse events (AEs).

### Risk of bias assessment

The risk of bias of involved RCT was analyzed following the Cochrane Collaboration’s tool [14]. And following domains were considered: allocation concealment, random sequence generation, blinding of participants and outcome assessment, incomplete outcome data, selective outcome reporting, and other bias [14]. If any domain was identified as high risk of bias, the study was classified as having high risk of bias. If every above domain was at low risk of bias, the study was classified as having low risk of bias. If there appears a domain with unclear risk of bias, then the study was classified as having unclear risk of bias.

### Outcomes

The primary outcomes were pain scores, including WOMAC pain score, VAS score, AUSCAN pain score, and NRS score. Secondary outcomes included treatment-related adverse events (AEs). AEs included the number of records AEs and number of participants with any adverse event or serious adverse event caused by side effects of medicine.

### Data analysis

We descriptively reported study data using means with 95% ranges for continuous variables, and counts and percentages for categorical variables. The studies we included contained high heterogeneity of baseline data, assessed values and scales. For the high heterogeneity in involved studies, we could only use descriptive statistics to present the results.

## Results

### Study selection

**Fig. 1:**
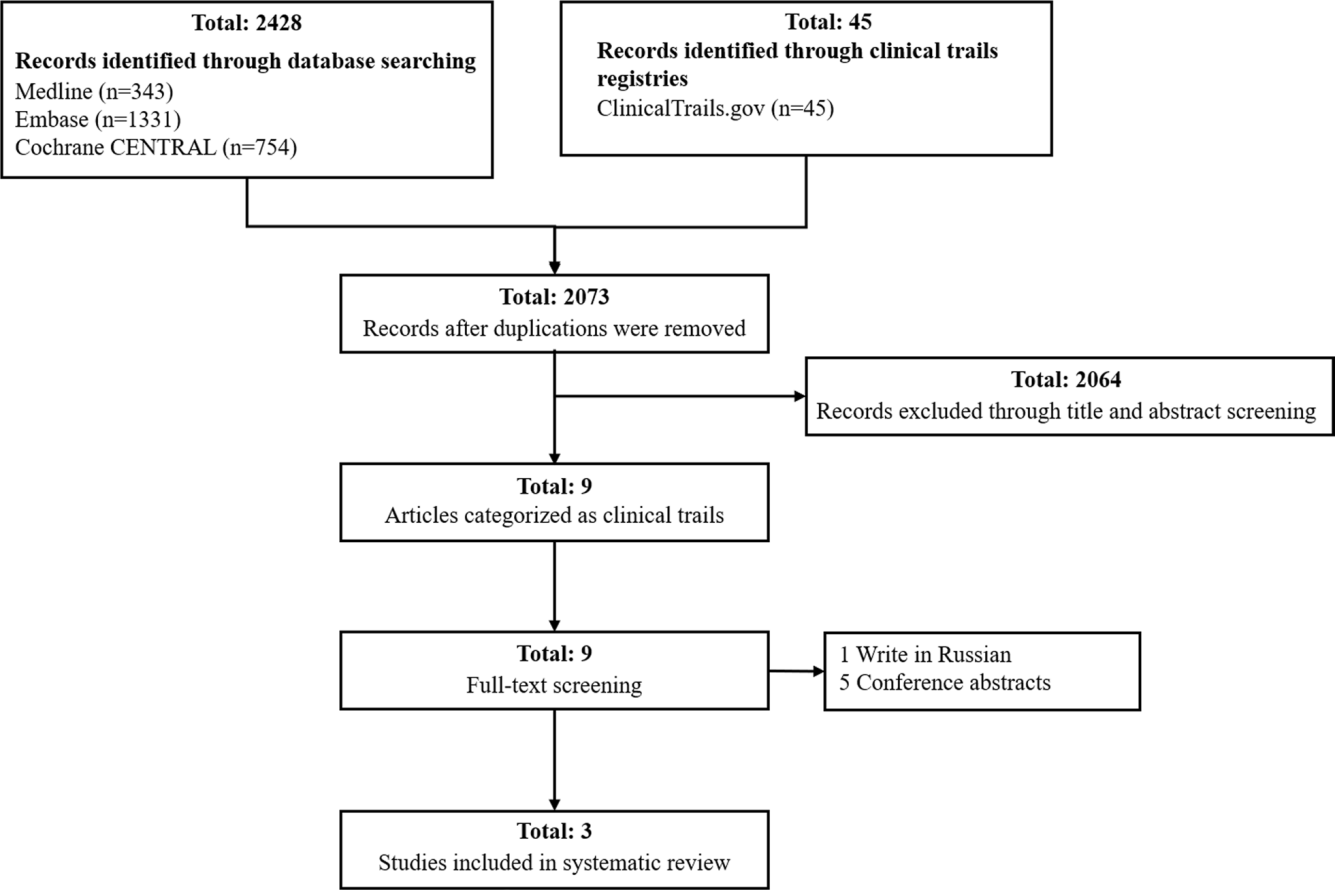
Study search strategy

In total, we included 2073 studies among these databases (343 studies in Medline, 1,331 studies in Embase, 754 studies in Cochrane CENTRAL, and 45 studies in ClinicalTrails) after removing duplications (Fig. 1). By screening title and abstract, we excluded 2,064 articles with exclusive criteria, and only 9 eligible literatures were moving to the next filtration step. After reading the full text of these studies, we excluded 5 conference abstracts and 1 research in Russian, then only 3 articles were included in our systematic review.

### Study characteristics

Three RCTs were included in our systematic review. All of them were published in last ten years. The study characteristics of these three RCTs are listed in Table 1. These three RCTs were all used double-blind study design. Two RCTs compared the efficacy of independent drugs in OA pain management, and one RCT compared combined use of medicines.

**Table 1:** Study characteristics

Study	Setting	Sample size	Study design	Follow up duration (weeks)
Sofat et al. [15]	St George’s University Hospitals, United Kingdom	65	Double-blind RCT comparing efficacy of pregabalin, duloxetine, and placebo	13
Ohtori et al. [17]	Chiba University Hospital, Japan	89	Open label RCT comparing efficacy of meloxicam, pregabalin, and combined usage of the two	4
Enteshari-Moghaddam et al. [18]	Ardabil University of Medical Sciences, Iran	150	Double-blind RCT comparing efficacy of gabapentin, duloxetine, and acetaminophen	12

### Participant characteristics

The characteristics of participant enrolled in the three RCTs were listed in Table 2. Sofat and colleagues [15] recruited 65 patients aged 40–75 years with hand OA diagnosed with American College of Rheumatology (ACR) criteria [16]. The participants had a NRS score over 5, and were on usual care including acetaminophen and/or NSAIDs. 22 patients were treated with pregabalin, 21 patients were treated with duloxetine, and 22 patients received placebo. The three groups have no statistic difference in age, female ratio, NRS score before treatment, and AUSCAN pain score. Ohtori and colleagues [17] recruited 89 patients with knee OA complaining knee pain at least one month and having X-ray of affected knee. All patients were assessed for the severity of knee OA with Kellgren-Lawrence (KL) grading system by X-ray imaging. 28 patients were treated with pregabalin, 31 patients were treated with meloxicam, and 30 patients were treated with pregabalin and meloxicam. There was no statistically significant difference in age, female ratio, knee OA severity, VAS score before treatment, and WOMAC pain score between the three groups. Enteshari-Moghaddam and colleagues [18] recruited 150 patients aged 45–75 years with knee OA classified KL 3 and 4 level. 50 patients were treated with gabapentin, 50 patients were treated with duloxetine, and 50 patients were in acetaminophen group. The participants characteristics were also similar among the three groups, including age, female ratio, knee OA severity, VAS score before treatment, and WOMAC pain score.

**Table 2:** Participants characteristics

Study	Nation	Female ratio	Age (years)	Affected joint	Disease duration	Severity of OA
Sofat et al. [15]	United Kingdom	0.80	40–75	Hand	3.5 ± 4.2 (mean ± SD) years	unclear
Ohtori et al. [17]	Japan	0.71	Unclear	Knee	35.5 ± 8.0 (mean ± SEM) months	KL 1: 24.7%; KL 2: 27.0%; KL 3: 34.8%; KL4, 13.5%
Moghaddam et al. [18]	Iran	0.73	45–75	Knee	Unclear	KL 2 to 3 with VAS ≥ 5 or WOMAC score ≥ 48

*SD* standard deviation, *SEM* standard error of the mean, *OA* osteoarthritis, *KL* Kellgren-Lawrence, *VAS* Visual analogue scale, *WOMAC* Western Ontario and McMaster Universities Arthritis Index

### Treatment details

The treatment details of included three RCTs were presented in Table 3. In Sofat et al. study, participants took one capsule of placebo or pregabalin or duloxetine at night in week one orally, then two capsules from week two to week ten, finally one capsule from week eleven to week twelve [15]. In Ohtori et al. study, participants in meloxicam group took meloxicam orally 30 min after breakfast, participants in pregabalin group took pregabalin orally before sleep, and combined medicine group took meloxicam 30 min after breakfast and pregabalin before sleep for four weeks [17]. In Enteshari-Moghaddam et al. study, participants took gabapentin or duloxetine or placebo for 3 months, and double the dose from week 3 [18].

**Table 3:** Treatment details

Study	GABA derivative	Usage and dose	Control medicine	Usage and dose
Sofat et al. [15]	pregabalin	150 mg q.n. in week 1, 11 and 12; 300 mg q,n. in week 2–10	Duloxetine	30 mg q.n. in week 1, 11 and 12; 60 mg q,n. in week 2–10
Ohtori et al. [17]	pregabalin	25 mg q.n. for four weeks	Meloxicam	10 mg q.d. for four weeks
Moghaddam et al. [18]	gabapendin	300 mg q.d. in week 1 and 2; 600 mg from week 3–month 3	Duloxetine	30 mg q.d. in week 1 and 2; 60 mg from week 3–month 3
			Acetaminophen	1000 mg q.d. in week 1 and 2; 2000 mg q.d. from week 3–month 3

*q.n.* quaque nocte, *q.d.* quaque die

### Efficacy and safety of GABA derivatives in OA pain management

All included studies reported OA pain relief with GABA derivatives treatment (Table 4). For hand OA, pregabalin reduced NRS score from 6.1 (95% CI: 5.4–6.7) to 3.4 (95% CI: 2.4–4.4) with a mean difference − 2.7 (95% CI: − 3.5 to − 1.9). The difference of mean difference of pregabalin effect on NRS score was statistically significant compared with placebo (− 0.9 (95% CI: − 0.2 to 0.2), P = 0.023). Meanwhile, pregabalin reduced AUSCAN pain score from 317.0 (95% CI: 280.8–353.1) to 176.5 (95% CI: 123.9–229.1) with a mean difference − 132.1 (95% CI: − 181.1 to − 82.9). The difference of mean difference of pregabalin effect on AUSCAN pain score was statistically significant compared with placebo (− 46.61 (95% CI: − 93.9 to 0.75), P = 0.008) [15]. For knee OA, pregabalin reduced VAS score from 5.0 ± 2.0 (mean ± standard error of the mean (SEM)) to 2.0 ± 2.2 (mean ± SEM) in four weeks. Meanwhile, pregabalin reduced WOMAC pain score from 12.2 ± 3.0 (mean ± SEM) to 6.6 ± 3.0 (mean ± SEM) in four weeks [17]. For knee OA, gabapentin reduced both VAS and WOMAC pain score in involved participants. Compared with acetaminophen {− 31.20 ± 12.58 [mean ± standard deviation (SD)]}, the mean difference of VAS score of gabapentin [− 63.36 ± 8.87 (mean ± SD)] was statistically significantly larger (P < 0.001). Meanwhile, compared with acetaminophen [− 50.30 ± 10.78 (mean ± SD)], the mean difference of WOMAC pain score of gabapentin [− 73.94 ± 12.79 (mean ± SD)] was statistically significantly larger (P < 0.001) [18].

**Table 4:** Efficacy of GABA derivatives in OA pain management

Study	Pain outcome measures	Summary results
Sofat et al. [15]	NRS, AUSCAN pain score	In intention-to-treat analysis and per protocol analysis, NRS and AUSCAN pain score decreased statistically significantly in pregabalin group compared with placebo group. Only in per protocol analysis NRS score decreased statistically significantly in duloxetine group compared with placebo group
Ohtori et al. [17]	VAS, WOMAC pain score	Pregabalin and meloxicam reduced VAS and WOMAC pain score of knee OA patient, but there was no statistically significant difference between pregabalin group and meloxicam group. However, a combined use of these two drugs was better than singular use
Moghaddam et al. [18]	VAS, WOMAC pain score	VAS and WOMAC pain score decreased statistically significantly in gabapentin group and duloxetine group compared with placebo group. However, no statistically significant difference was found in gabapentin group and duloxetine group

*NRS* Numerical Rating Scale, *AUSCAN* Australian and Canadian Hand Osteoarthritis Index, *VAS* Visual analogue scale, *WOMAC* Western Ontario and McMaster Universities Arthritis Index

Number and subtypes of recorded treatment-related AEs owing to side effects of GABA derivatives were summarized in Table 5. Sofat et al. reported 55 recorded treatment-related AEs in pregabalin group, mainly including nervous system disorder and mental disturbance [15]. Ohtori et al. reported no treatment-related AE in their study [17]. Enteshari-Moghaddam et al. reported 9 recorded treatment-related AEs in 9 patients (18%) in gabapentin group [18].

**Table 5:** Safety of GABA derivatives

Study	Number of recorded AEs	Type of recorded
Sofat et al. [15]	55	Cardiovascular 3; Digestive 7; Endocrine 1; Mental 9; Nervous system 28; Ophthalmological 4; Respiratory 2; Genitourinary 1
Ohtori et al. [17]	0	No record
Moghaddam et al. [18]	9	Dry mouth 5; Drowsiness 2; Fatigue in 2

### Quality of evidence

There lacks available peer-reviewed literature and rigorous study design on this topic. In that case, the risk of confounding is moderate to severe for included studies. Bias for selection, performance, detection, attrition, and reporting of the included RCTs are lists in Table 6.

**Table 6:** Bias assessment in three RCTs

Study	Selection bias		Performance bias	Detection bias	Attrition bias	Reporting bias
	Random sequence generation	Allocation concealment	Blinding of participants and personnel	Blinding of outcome assessment	Incomplete outcome data	Selective reporting
Sofat et al. [15]	Randomization was implemented through sequentially numbered container	Not mentioned	Blind to participants	Blind to investigators	13/65 participants quit the study	All prespecified outcomes were reported
Judgement	Low risk	Unclear risk	Low risk	Low risk	High risk	Low risk
Ohtori et al. [17]	Patients were randomized with the minimization method	Not mentioned	Not mentioned	Blind to observers for radiographic evaluation of Knee OA	No participant quit the study	All prespecified outcomes were reported
Judgement	Low risk	Unclear risk	Unclear risk	Unclear risk	Low risk	Low risk
Moghaddam et al. [18]	Patients were randomized by random number	Not mentioned	Blind to participants	Blind to investigators	No participant quit the study	All prespecified outcomes were reported
Judgement	Low risk	Unclear risk	Low risk	Low risk	Low risk	Low risk

## Discussion

This systematic review summarized the published researches focused on GABA derivatives use in OA pain relief. And we found that GABA derivatives were effective in OA pain control and the safety of GABA derivatives utilization in OA patients was quite satisfactory [15, 17, 18]. In our results, the analgesic effect of pregabalin on OA pain relief was even better than duloxetine and NSAIDs. Meanwhile, the analgesic effect of gabapentin on OA pain control was similar to duloxetine but better than NSAIDs.

To explain the results in our study, we should first focus on the pain generation in OA patients. As mentioned above, OA pain mechanisms can be mainly divided into three groups: (a) nociceptive pain; (b) neuropathic pain; (c) central pain. For nociceptive pain, the pain is caused by directly and indirectly activation of peripheral nociceptors (sensory receptors for painful stimuli) through multiple cytokines, including NGF, tumor necrosis factor (TNF), and prostaglandins, induced by inflammation in OA joint [19]. Eicosanoid, prostaglandin E2 (PGE2), produced by immune and non-immune cells in OA joint, is another activator of nociceptors, which leads to nociceptive pain in OA joint [20]. Furthermore, as nociceptors are stimulated, they produce calcitonin gene-related peptide (CGRP) and substance P, which in turn enriches inflammatory cells and descends nociceptive threshold, and aggravates OA pain [21]. For neuropathic pain, the pain is caused by sensory nerve fiber injury owing to the increased concentration of the lipid mediator lysophosphatidic acid in OA synovial fluid, which can demyelinate peripheral nerves, and do harm to peripheral nerve fibers [22]. In damaged sensory nerve fibers, numerous abnormal impulses are generated from different sites of the neuron including neuroma and axon, owing to increased amount of voltage-gated Na + channels, resulting in more frequent spontaneous discharge in neurons [23]. When the injured neuron begins to regenerate, it stretches out its nerve fibers and builds new connections with adjacent neurons, which were not linked previously, resulting in abnormal nervous conjunction [24]. For central pain, continuous stimulation of peripheral nervous system with OA strike forces peripheral sensory neurons to produce pain-related neurotransmitters including glutamate, substance P, NGF, and CGRP constantly, resulting in pain hypersensitivity in brain [25]. In that case, the descending pain facilitation pathways from central to peripheral are enhanced by facilitatory neurotransmitters secreted by central nervous system, including glutamate, aspartate, and serotonin. Meanwhile, the descending pain inhibition pathways from central to peripheral are weakened owing to lack of inhibitory neurotransmitters secreted by central nervous system, including serotonin, norepinephrine, GABA, and opioids [6].

Anti-inflammatory drugs such as NSAIDs can decrease inflammatory cytokines, and release nociceptive pain, but have a slight effect on neuropathic and central pain [26]. Pregabalin was proved to be efficacious to treat neuropathic pain. Although pregabalin is GABA derivative, sufficient evidences have proved it has no agonist-like effect on GABA receptors, which indicates pregabalin releasing pain through other mechanisms [27]. Pregabalin can bind and block α2$\delta$ subunit of sensory neurons and depress the expression of neuron terminal channel, resulting in excitability reduction of these neurons, which releases neuropathic pain [28]. Meanwhile, pregabalin can specifically bind α2$\delta$ subunit of Cav channels in central nervous system, and reduce central excitability, resulting in a central pain relief [29]. Pregabalin also inhibits GABA release in locus coeruleus (LC) to rescue the inhibition of noradrenergic descending pathways after nerve damage, and reduce the excitability of nociceptive neurons [30].

Gabapentin, as another GABA derivative, has the similar effect on chronic pain control compared with pregabalin. Gabapentin can also bind α2$\delta$ subunit of peripheral and central neurons. Gabapentin may inhibit 5-HT3 receptors, a descending pain facilitation pathway, and release neuropathic pain [31]. Owing to a dense α2$\delta$ subunit expression in brainstem, gabapentin can block the descending modulatory pain inputs activated by periaqueductal gray matter, and release central pain [32]. Gabapentin can also reduce presynaptic GABA release in LC and release central pain [33]. At the same time, gabapentin stimulates dopamine (DA) secretion in nucleus accumbens (NAc) through opioid signaling pathway, and reduces central pain [34].

Our study has several strengths and limitations. One advantage of our study is using systematical approach to identify all published literatures of GABA derivatives usage in OA pain management. However, only a few literatures focused on this term, and we only included three eligible RCTs after literature screening and filtrating. This limits the strength of the evidence in our study and may cause deviation of the results. Another limitation of this study is that the heterogenicity of these included RCTs was too strong, owing to the differences in affected OA joints, participants characteristics, usage of medicine, duration of treatment, and pain assessment. In that case, we could only present our results with description approach.

All in all, combined with the analgesic mechanisms and current clinical practice in OA pain control, GABA derivatives are very likely to be an effective OA analgesics and analgesic adjuvants. However, few studies have focused on this topic, the evidence is still insufficient. Future studies are demanded to further prove the analgesic efficacy of GABA derivatives as OA analgesics and analgesic adjuvants.

## Conclusions

Our systematic review comprehensively described the efficacy and safety of GABA derivatives including pregabalin and gabapentin use in OA pain management. However, owing to small number of included clinical trials, extreme heterogeneity of involved participants and lack of large sample size and/or multi-center study, the accurate efficacy of GABA derivatives on OA pain management is still elusive. Although, according to the present studies, GABA derivatives seem to be effective and safe in OA pain management, more future studies are commanded to further explore the utilization of GABA derivatives in OA pain control.

## Supplementary Information

**Additional file 1.** Search strategy.

## Data Availability

All data generated or analyzed during this study are included within this published article.

## Abbreviations

OA: Osteoarthritis
GABA: Gamma-aminobutyric acid
RCT: Randomized controlled trial
NRS: Numerical rating scale
AUSCAN: Australian/Canadian Osteoarthritis Hand Index
VAS: Visual analogue scale
WOMAC: Western Ontario and McMaster Universities Arthritis Index
AE: Adverse event
NSAIDs: Nonsteroidal anti-inflammatory drugs
NGF: Nerve growth factor
Na: Sodium
Cav: Voltage-gated calcium
KL: Kellgren-Lawrence
TNF: Tumor necrosis factor
PGE2: Prostaglandin E2
CGRP: Calcitonin gene-related peptide
LC: Locus coeruleus
NAc: Nucleus accumbens
SD: Standard deviation
SEM: Standard error of the mean
q.n.: Quaque nocte
q.d.: Quaque die

## References

[1] Hunter DJ, Bierma-Zeinstra S (2019). Osteoarthritis. The Lancet.

[2] Neogi T (2013). The epidemiology and impact of pain in osteoarthritis. Osteoarthr Cartil.

[3] Liu A, Kendzerska T, Stanaitis I, Hawker G (2014). The relationship between knee pain characteristics and symptom state acceptability in people with knee osteoarthritis. Osteoarthr Cartil.

[4] Malfait A, Schnitzer T (2013). Towards a mechanism-based approach to pain management in osteoarthritis. Nat Rev Rheumatol.

[5] Dimitroulas T, Duarte R, Behura A, Kitas G, Raphael J (2014). Neuropathic pain in osteoarthritis: a review of pathophysiological mechanisms and implications for treatment. Semin Arthritis Rheum.

[6] Lluch E, Torres R, Nijs J, Van Oosterwijck J (2014). Evidence for central sensitization in patients with osteoarthritis pain: a systematic literature review. Eur J Pain.

[7] Osani M, Vaysbrot E, Zhou M, McAlindon T, Bannuru R (2020). Duration of symptom relief and early trajectory of adverse events for oral nonsteroidal antiinflammatory drugs in knee osteoarthritis: a systematic review and meta-analysis. Arthritis Care Res.

[8] He W, Kuang M, Zhao J, Sun L, Lu B, Wang Y (2017). Efficacy and safety of intraarticular hyaluronic acid and corticosteroid for knee osteoarthritis: a meta-analysis. Int J Surg (Lond, Engl).

[9] Yang S, Huang Y, Ye Z, Li L, Zhang Y (2020). The efficacy of nerve growth factor antibody for the treatment of osteoarthritis pain and chronic low-back pain: a meta-analysis. Front Pharmacol.

[10] Uchio Y, Enomoto H, Alev L, Kato Y, Ishihara H, Tsuji T (2018). A randomized, double-blind, placebo-controlled Phase III trial of duloxetine in Japanese patients with knee pain due to osteoarthritis. J Pain Res.

[11] Rock D, Kelly K, Macdonald R (1993). Gabapentin actions on ligand- and voltage-gated responses in cultured rodent neurons. Epilepsy Res.

[12] Gee N, Brown J, Dissanayake V, Offord J, Thurlow R, Woodruff G (1996). The novel anticonvulsant drug, gabapentin (Neurontin), binds to the alpha2delta subunit of a calcium channel. J Biol Chem.

[13] Chiechio S, Zammataro M, Caraci F, Rampello L, Copani A, Sabato AF (2009). Pregabalin in the treatment of chronic pain: an overview. Clin Drug Investig.

[14] Higgins JP, Altman DG, Gotzsche PC, Juni P, Moher D, Oxman AD (2011). The Cochrane Collaboration's tool for assessing risk of bias in randomised trials. BMJ..

[15] Sofat N, Harrison A, Russell MD, Ayis S, Kiely PD, Baker EH (2017). The effect of pregabalin or duloxetine on arthritis pain: a clinical and mechanistic study in people with hand osteoarthritis. J Pain Res.

[16] Altman R, Alarcón G, Appelrouth D, Bloch D, Borenstein D, Brandt K (1990). The American College of Rheumatology criteria for the classification and reporting of osteoarthritis of the hand. Arthritis Rheum.

[17] Ohtori S, Inoue G, Orita S, Takaso M, Eguchi Y, Ochiai N (2013). Efficacy of combination of meloxicam and pregabalin for pain in knee osteoarthritis. Yonsei Med J.

[18] Enteshari-Moghaddam A, Azami A, Isazadehfar K, Mohebbi H, Habibzadeh A, Jahanpanah P (2019). Efficacy of duloxetine and gabapentin in pain reduction in patients with knee osteoarthritis. Clin Rheumatol.

[19] Schaible H (2014). Nociceptive neurons detect cytokines in arthritis. Arthritis Res Ther.

[20] Pinho-Ribeiro F, Verri W, Chiu I (2017). Nociceptor sensory neuron-immune interactions in pain and inflammation. Trends Immunol.

[21] Ji RR, Chamessian A, Zhang YQ (2016). Pain regulation by non-neuronal cells and inflammation. Science.

[22] Fu K, Robbins SR, McDougall JJ (2018). Osteoarthritis: the genesis of pain. Rheumatology (Oxford)..

[23] Cohen SP, Mao J (2014). Neuropathic pain: mechanisms and their clinical implications. BMJ..

[24] Komori K, Nonaka T, Okada A, Kinoh H, Hayashita-Kinoh H, Yoshida N (2004). Absence of mechanical allodynia and Abeta-fiber sprouting after sciatic nerve injury in mice lacking membrane-type 5 matrix metalloproteinase. FEBS Lett.

[25] Mease PJ, Hanna S, Frakes EP, Altman RD (2011). Pain mechanisms in osteoarthritis: understanding the role of central pain and current approaches to its treatment. J Rheumatol.

[26] Conaghan PG, Cook AD, Hamilton JA, Tak PP (2019). Therapeutic options for targeting inflammatory osteoarthritis pain. Nat Rev Rheumatol.

[27] Patel R, Dickenson AH (2016). Mechanisms of the gabapentinoids and alpha 2 delta-1 calcium channel subunit in neuropathic pain. Pharmacol Res Perspect..

[28] Colloca L, Ludman T, Bouhassira D, Baron R, Dickenson A, Yarnitsky D (2017). Neuropathic pain. Nat Rev Disease Primers.

[29] Stahl S, Porreca F, Taylor C, Cheung R, Thorpe A, Clair A (2013). The diverse therapeutic actions of pregabalin: Is a single mechanism responsible for several pharmacological activities?. Trends Pharmacol Sci.

[30] Takeuchi Y, Takasu K, Ono H, Tanabe M (2007). Pregabalin, S-(+)-3-isobutylgaba, activates the descending noradrenergic system to alleviate neuropathic pain in the mouse partial sciatic nerve ligation model. Neuropharmacology.

[31] Suzuki R, Dickenson A (2006). Differential pharmacological modulation of the spontaneous stimulus-independent activity in the rat spinal cord following peripheral nerve injury. Exp Neurol.

[32] Hayashida K, Eisenach J (2008). Multiplicative interactions to enhance gabapentin to treat neuropathic pain. Eur J Pharmacol.

[33] Yoshizumi M, Parker RA, Eisenach JC, Hayashida K (2012). Gabapentin inhibits γ-amino butyric acid release in the locus coeruleus but not in the spinal dorsal horn after peripheral nerve injury in rats. Anesthesiology.

[34] Bannister K, Qu C, Navratilova E, Oyarzo J, Xie J, King T (2017). Multiple sites and actions of gabapentin-induced relief of ongoing experimental neuropathic pain. Pain.

