# Supplementary material for: Pharmacological use of gamma-aminobutyric acid derivatives in osteoarthritis pain management: a systematic review
Source: BMC Rheumatol. 2022 May 11;6:28. doi: 10.1186/s41927-022-00257-z (PMC9092798; doi:10.1186/s41927-022-00257-z)
Supplement: Supplementary file 1 — Additional file 1. Search strategy. [file 41927_2022_257_MOESM1_ESM.pdf]

Medline

'osteoarthritis' AND 'pregabalin' 58 results
'osteoarthritis' AND 'gabapentin' 54 results
'osteoarthritis' AND 'vigabatrin' 1 results
'osteoarthritis' AND 'baclofen' 6 results
'osteoarthritis' AND 'gamma-Aminobutyric Acid' 67 results
'osteoarthritis' AND 'gabapentinoids' 10
'osteoarthritis' AND 'anticonvulsants' 147
total 343

Cochrane library

Search Name:OA and GABA
Date Run: 03/10/2021 01:45:46
Comment:

ID Search Hits
#1 MeSH descriptor: [Osteoarthritis] this term only 4380
#2 osteoarthr* OR degenera*:ti,ab,kw 29694
#3 #1 or #2 29694
#4 MeSH descriptor: [Pregabalin] this term only 855
#5 MeSH descriptor: [Gabapentin] this term only 883
#6 MeSH descriptor: [Baclofen] this term only 264
#7 MeSH descriptor: [Vigabatrin] this term only 133
#8 MeSH descriptor: [gamma-Aminobutyric Acid] this term only 1289
#9 Gamma* OR Amin* OR GABA:ti,ab,kw 46983
#10 #8 OR #9 46983
#11 isobutyl OR Lyrica OR methylhexanoic OR 1008 OR pregabalin:ti,ab,kw 4310
#12 #4 OR #11 4310
#13 Convalis OR gabapentin OR Neurontin OR cyclohexaneacetic:ti,ab,kw 2705
#14 #5 OR #13 2705
#15 Baclofène OR Liorésal OR Lioresal OR 34647 OR genpharm OR baclofen OR Chlorophenyl OR Baclophen OR PCP-GABA:ti,ab,kw 3360
#16 #6 OR #15 3360
#17 Vinyl OR Vigabatrin OR Sabrilex OR Sabril:ti,ab,kw 737
#18 #7 OR #17 737
#19 #10 OR #12 OR #14 OR #16 OR #18 55339
#20 #3 AND #19 752
#21 #1 AND {OR #4-#8} 16
#22 gabapentinoids 79
#23 #22 AND #3 1
#24 MeSH descriptor: [Anticonvulsants] this term only 2474
#25 #24 AND #3 10

clinicaltrail+meta+systematic review 752-9(protocol)+11=754

EMbase

'osteoarthritis' AND 'gamma-Aminobutyric Acid' 14
'osteoarthritis' AND 'vigabatrin' 7
'osteoarthritis' AND 'Sabril' 1
'osteoarthritis' AND 'Pregabalin' 439
'osteoarthritis' AND 'gabapentin' 554
'osteoarthritis' AND 'Neurontin' 27
'osteoarthritis' AND 'Baclofen' 82
'osteoarthritis' AND 'Lioresal' 8
'osteoarthritis' AND 'lyrica' 30
'osteoarthritis' AND 'gabapentinoid*' 24
'osteoarthritis' AND 'anticonvulsant*' 145
total 1331

ClinicalTrail.gov

'osteoarthritis' AND 'pregabalin' 8 results
'osteoarthritis' AND 'gabapentin' 11 results
'osteoarthritis' AND 'vigabatrin' 0 results
'osteoarthritis' AND 'baclofen' 2 results
'osteoarthritis' AND 'gamma-Aminobutyric Acid' 0 results
'osteoarthritis' AND 'gabapentinoids' 1
'osteoarthritis' AND 'anticonvulsants' 23
total 45
